# Whole-Volume ADC Histogram Analysis in Parotid Glands to Identify Patients with Sjögren’s Syndrome

**DOI:** 10.1038/s41598-019-46054-6

**Published:** 2019-07-03

**Authors:** Chen Chu, Qianqian Feng, Huayong Zhang, Yun Zhu, Weibo Chen, Jian He, Lingyun Sun, Zhengyang Zhou

**Affiliations:** 1 Department of Radiology, Nanjing Drum Tower Hospital, The Affiliated Hospital of Nanjing University Medical School, Nanjing, 210008 China; 2 Department of Rheumatology, Nanjing Drum Tower Hospital, The Affiliated Hospital of Nanjing University Medical School, Nanjing, 210008 China; 3 Philips Healthcare, Shanghai, 200233 China

**Keywords:** Autoimmune diseases, Autoimmune diseases

## Abstract

At present, no gold standard for diagnosing Sjögren’s syndrome (SS) is available in clinical practice. The 2002 American–European Consensus Group classification criteria are used to diagnose SS. Clinically, it is challenging to distinguish patients with SS from suspected patients undergoing different therapies. A total of 52 patients with SS and 24 patients suspected of having the disease prospectively underwent 3.0-T magnetic resonance (MR) scanning, including diffusion-weighted imaging (*b* = 0 and 1000 s/mm^2^). The whole-volume apparent diffusion coefficient (ADC) histogram analysis generated ADC_mean_, skewness, kurtosis, and entropy values from bilateral parotid glands. Continuous variables were compared using an independent two-sample *t* test, and categorical variable compared using the Fisher’s test between the two groups. Receiver operating characteristic (ROC) analysis was used to evaluate the diagnostic performance of the indexes. Fisher’s tests demonstrated that some clinical indexes and MR morphology grades differed significantly between patients with SS and patients suspected of having the disease (all *P* ≤ 0.001). The parotid entropy value of patients with SS was significantly higher than that of patients suspected of having the disease (*P* < 0.001). Among MR parameters, entropy combined with kurtosis performed the best in differentiating patients with SS from those suspected of having SS (area under the ROC curve = 0.955). A whole-volume ADC histogram analysis might provide a series of parameters that reflect tissue characteristics.

## Introduction

Sjögren’s syndrome (SS) is a chronic autoimmune systematic disease, causing direct damage to exocrine glands. At present, a gold standard diagnostic assay for SS is not available in clinical practice. According to the 2002 American–European Consensus Group (AECG) classification criteria^1^, the diagnosis of SS is based on the results of serological tests, salivary tests, ocular tests, and histopathology of the labial gland. Imaging the injury to the salivary glands is critical for diagnosing SS.

Although X-ray sialography and sialoscintigraphy have been added to the AECG criteria as imaging modalities, X-ray sialography is invasive^2,3^ and technetium-99m pertechnetate scintigraphy results in exposure to radionuclides^4,5^. Ultrasonography and computed tomography (CT) provide qualitative evaluation; however, CT involves the use of ionizing radiation^6,7^. Magnetic resonance (MR) imaging (MRI) is increasingly used because it is noninvasive and does not require radiation exposure^8,9^. However, MRI, including MR sialography, proved to be insensitive to early injury of the parotid glands^10,11^.

Therefore, various functional MR sequences were applied to evaluate parotid injury in SS, such as diffusion-weighted imaging (DWI)^12,13^, dynamic contrast enhancement (DCE)^14^, and intravoxel incoherent motion (IVIM)^15^. DWI has been the most widely used modality because of its ability to noninvasively quantify water molecular diffusion by calculating apparent diffusion coefficient (ADC) values^16^. Diagnosing SS using DWI has been investigated by several groups. Regier *et al*. demonstrated that ADC values increase in early-stage SS and decrease in advanced-stage SS, based on MR sialography grading. They declared that DWI could display functional changes in the parotid gland affected by SS and might be a useful tool for differentiating between the early and advanced disease stages if combined with MR sialography^17^. Ding *et al*. reported that when using multiple small regions of interest (ROIs), the signal intensity ratio (SIR) values of the parotid versus spinal cord and parotid ADC values in DWI differed significantly between SS patients and non-SS patients (patients suspected of having SS + healthy volunteers)^12^. The receiver operating characteristic (ROC) analysis showed that ADC values are less diagnostically effective than SIR values. Multiple small ROIs could better reflect the heterogeneity of the parotid glands, presenting a higher diagnostic value compared with traditional ROI. However, Ding *et al*. did not conduct a ROC analysis for patients with SS and patients suspected of having SS, probably due to the small sample size (only five patients were suspected of having SS)^12^. Xu *et al*. also confirmed the use of ADC values in diagnosing a patient with early-stage SS^13^.

ADC histogram analysis could provide many parameters reflecting tissue characteristics, for instance, hypoxia, angiogenesis, and cellular proliferation in cancer lesions^18^, or edema and neovascularization in inflammatory diseases^19,20^. The histological analysis of early parotid injury typically reveals a loss of normal gland architecture and lymphocytic and plasma cell infiltration^21,22^. ADC texture analysis was used in our study to evaluate the disease activity of patients with primary SS using MRI and clinical and laboratory indicators^23^. To date, whole-volume ADC histogram analysis had never been reported in diagnosis of SS.

Hence, his study aimed to explore the use of parotid whole-volume ADC histogram analysis to distinguish patients suspected of having SS from those with the disease.

## Results

### Parotid MR morphology grades

The MR morphology grades of bilateral parotid glands were found to be consistent in all patients and were valued at 0 for patients suspected of having SS. The MR morphological grades were 0, 1, 2, and 3 in 21 (40.4%), 11 (21.2%), 12 (23.1%), and 8 (15.4%) patients with SS, respectively. The MR morphology of the bilateral parotid glands were consistently grade 0 in all volunteers.

### ADC values obtained from one ROI of parotid gland

There were no significant differences of ADC values based on one ROI between bilateral parotid glands. Hence, we calculated average ADC values of bilateral parotid glands as the final one for each patient. Parotid ADC values from volunteers were significantly higher than those of patients with SS or patients suspected of having SS (*P* = 0.008 and 0.039, respectively). However, no significant differences were observed between patients with SS and patients suspected of having SS (*P* = 0.625).

### Whole-volume ADC histogram analysis of parotid glands

There were no significant differences of whole-volume ADC histogram parameter values between bilateral parotid glands. Hence, we calculated average ADC histogram parameter values of the bilateral glands for each patient.

**Figure 1:**
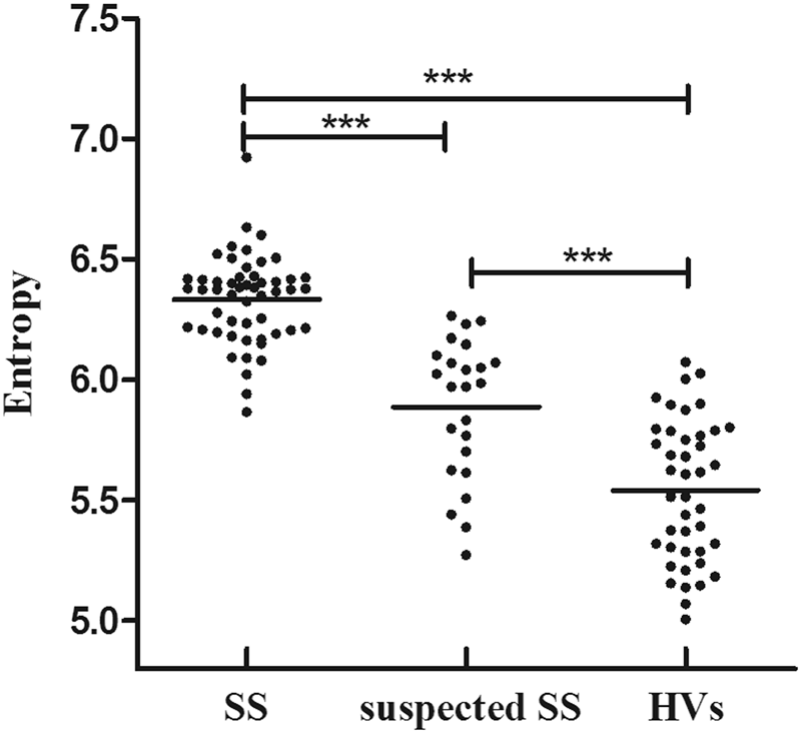
Scatterplots of parotid apparent diffusion coefficient (ADC) entropy in patients with SS, patients suspected of having SS, and healthy volunteers. ***P < 0.001.

Parotid entropy values of patients with SS were significantly higher than those of patients suspected of having SS (*P* < 0.001). The parotid entropy values of healthy volunteers were significantly lower than those of patients with SS (*P* < 0.001), and the entropy values of healthy volunteers were significantly lower than those of patients suspected of having SS (*P* < 0.001). Scatterplots of entropy in patients with SS, patients suspected of having SS, and healthy volunteers are shown in Fig. 1.

No significant differences were observed in the ADC_mean_, skewness, and kurtosis values between the two groups. Detailed results are shown in Table 1. The parotid ADC_mean_ and skewness values of volunteers were significantly higher than those of patients with SS (*P* = 0.001 and 0.019, respectively). The parotid kurtosis values of healthy volunteers were significantly lower than those of patients with SS (*P* = 0.004). The parotid ADC_mean_, skewness, and kurtosis values of volunteers were significantly higher than those of patients suspected of having SS (*P* = 0.003, 0.001, and <0.001, respectively).

**Table 1:** Diagnostic performance of various indexes in differentiating patients with Sjögren’s syndrome (SS) from patients suspected of having SS.

Parameters	Cutoff value	Sensitivity	Specificity	Accuracy	AUC	*P*
Anti-SSA and/or anti-SSB antibody	+	75.0%	62.5%	71.1%	0.688	0.009^*^
Ocular tests	+	40.4%	66.7%	48.7%	0.359	0.566
X-ray sialography	+	73.1%	58.3%	68.4%	0.657	<0.029^*^
Lip biopsy	+	34.6%	91.7%	52.6%	0.631	0.067
MR morphology grade	Grades 1–3	59.6%	100.0%	72.4%	0.798	<0.001^*^
ADC (×10^−6^ mm^2^/s)	<647.4	25.0%	87.5%	44.7%	0.522	0.759
ADC_mean_ (×10^−6^ mm^2^/s)	<669.6	28.8%	87.5%	47.4%	0.534	0.639
Skewness	<0.571	40.4%	87.5%	47.4%	0.589	0.215
Kurtosis	>4.930	40.0%	81.5%	53.7%	0.625	0.085
Entropy	>6.15	88.5%	83.3%	86.8%	0.924	<0.001^*^

Note, AUC, Area under the receiver operating characteristic curve; ^*^P < 0.05.

The parotid kurtosis and entropy values of patients with SS of grade 0 were significantly higher than those of patients suspected of having SS (*P* = 0.036 and <0.001, respectively).

The Spearman correlation test showed that the parotid ADC and ADC_mean_ correlated negatively with the MR morphology grade (*r* = −0.279, *P* = 0.039; *r* = −0.292, *P* = 0.035), and that parotid entropy correlated positively with MR morphology grade (*r* = 0.505, *P* < 0.001). Parotid skewness and kurtosis showed no significant correlation with the MR morphology grade (*r* = 0.181, *P* = 0.199; *r* = −0.157, *P* = 0.267, respectively).

**Figure 2:**
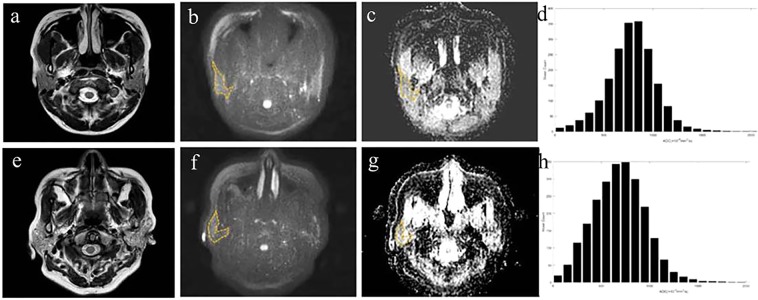
(**a**–**d**) T2WI, DWI, ADC map, and corresponding histograms of the bilateral parotid glands in a 26-year-old female suspected of having SS. The MR morphology grade, ADC, ADC_mean_, skewness, kurtosis, and entropy were 0, 815.1 × 10^−6^ mm^2^/s, 887.9 × 10^−6^ mm^2^/s, 0.358, 2.943, and 6.025, respectively. (**e**–**h**) The T2WI, DWI, ADC map, and corresponding histograms of the bilateral parotid glands in a 32-year-old female patient with SS. The MR morphology grade, ADC, ADC_mean_, skewness, kurtosis, and entropy were 1, 755.4 × 10^−6^ mm^2^/s, 817.9 × 10^−6^ mm^2^/s, 0.269, 4.031, and 6.353, respectively. Note the dashed lines covering the edge of right parotid gland.

Figure 2 shows representative T2WI, DWI (*b* = 1000 s/mm^2^), corresponding ADC maps, and ADC histograms of both groups.

### Diagnostic performance

ROC analysis showed that entropy presented the largest area under the ROC curve (AUC) of 0.924 (Table 1). The goodness-of-fit Hosmer–Lemeshow test was used to assess multivariate model calibration (*P* = 0.906). Kurtosis combined with entropy yielded a sensitivity value of 86.5%, a specificity value of 91.7%, an accuracy level of 88.2%, and an AUC value of 0.955 (*P* < 0.001) (Table 2). Based on the McNeil test, kurtosis combined with entropy showed a significantly higher AUC than any single index (all *P* < 0.05).

**Table 2:** Multivariate model for distinguishing patients with Sjögren’s syndrome (SS) from patients suspected of having SS.

	Log OR	SE	OR	*P*
Kurtosis	1.159	0.491	5.580	0.018
Entropy	12.498	3.301	14.336	<0.001

Note, Log OR, Logarithm of odds ratio; OR, odds ratio; SE, standard deviation.

With a cutoff value of 4.825, parotid kurtosis could differentiate patients with SS of grade 0 from patients suspected of having SS with a sensitivity of 57.1%, a specificity of 83.3%, and an accuracy of 71.1% (AUC = 0.683). With a cutoff value of 6.075, parotid entropy could differentiate patients with SS of grade 0 from patients suspected of having SS with a sensitivity of 85.7%, a specificity of 75.0%, and an accuracy of 80.0% (AUC = 0.839).

### Intraobserver and interobserver agreement of MR parameters

ADC and all ADC histogram parameters of parotid glands showed excellent intraobserver and interobserver agreements with intraclass correlation coefficients (ICCs) of 0.898–0.986 (Table 3). The interobserver agreement for evaluating the MR morphology grade was also excellent in the two groups (kappa coefficient = 1.000 and 0.950, respectively).

**Table 3:** Intra- and interobserver agreement of apparent diffusion coefficient (ADC) value and ADC histogram parameters in patients with Sjögren’s syndrome (SS) and patients suspected of having SS.

Parameter	Patients suspected of having SS		Patients with SS	
	Intraobserver	Interobserver	Intraobserver	Interobserver
ADC	0.947 (0.902–0.968)	0.952 (0.911–0.972)	0.898 (0.882–0.925)	0.902 (0.890–0.928)
ADC_mean_	0.982 (0.955–0.991)	0.986 (0.965–0.993)	0.908 (0.899–0.935)	0.911 (0.902–0.921)
Skewness	0.912 (0.905–0.920)	0.917 (0.907–0.925)	0.899 (0.862–0.922)	0.932 (0.882–0.961)
Kurtosis	0.921 (0.909–0.973)	0.941 (0.923–0.981)	0.902 (0.886–0.942)	0.915 (0.897–0.922)
Entropy	0.952 (0.931–0.976)	0.935 (0.917–0.967)	0.926 (0.907–0.942)	0.937 (0.912–0.949)

Note, Data in parentheses are 95% confidence intervals.

## Discussion

In this study, the differences in the whole-volume ADC histogram analysis on the bilateral parotid glands were compared between patients with SS and patients suspected of having SS. The study established the role of ADC histogram parameters in differentiating SS in the suspected group for the first time. It could help clinicians make different treatment plans.

Parotid ADC values were compared among patients with SS, patients suspected of having SS, and healthy volunteers. The ADC values of patients with SS were significantly lower than those of healthy volunteers, probably due to damage to the parotid glands. Nevertheless, no significant difference was found between patients with SS and patients suspected of having SS. Ding *et al*.^12^ also reported that the ADC value could hardly differentiate patients with SS from non-SS patients (patients suspected of having SS + volunteers). In clinical practice, some patients suspected of SS may be diagnosed with dry mouth or other symptoms related to xerophthalmia and xerostomia; also, some of them have immunity-related complications, such as undifferentiated connective tissue disease or early-stage SS. Differentiation between patients with SS and patients suspected of having SS can prevent overtreatment of patients.

Significant differences were observed in the ADC_mean_, skewness, kurtosis, and entropy values based on ADC histogram and texture analysis between patients with SS and healthy volunteers. Only the entropy differed significantly between patients with SS and patients suspected of having SS. Kurtosis describes the sharpness and tails of ADC distribution, and skewness characterizes the degree of asymmetry from the normal distribution^18,24^. However, our study did not detect significant differences of skewness and kurtosis between patients with SS and patients suspected of having SS. Entropy is a measure of the disordered state of the system in statistical thermodynamics. The ADC entropy reflects the heterogeneity of various tumors, such as those of lung, breast, esophageal and colorectal cancers, which is closely related to tumor malignancy, patient survival, and prognosis^25–28^. The ADC entropy can also be used in evaluating inflammatory diseases. For instance, Makanyanga *et al*. indicated that the entropy could reflect bowel activity in Crohn’s disease through image heterogeneity and complexity^19^. Sidhu *et al*. suggested that ADC entropy might partially reflect changes in perfusion difference^29^. Perfusion changes in the parotid glands of patients with SS have been demonstrated in studies using DCE-MRI^14^ and IVIM^15^. The ADC entropy of SS patients was significantly higher than that of patients suspected of having SS and healthy volunteers. It was speculated that the microstructural and perfusion heterogeneity of patients with SS was more pronounced than that of patients suspected of having SS and healthy volunteers.

The parotid glands of patients with SS showed characteristic morphological changes on MRI^8,11^. According to the grading system of Makula *et al*.^11^, patients with SS who have no morphological changes in the parotid glands were graded as 0. The present study found that both kurtosis and entropy could differentiate patients with grade 0 SS from patients suspected of having SS. Additionally, the diagnostic performance of entropy was relatively better, providing a new way to address the clinical challenge.

This study found that the parotid ADC_mean_ correlates negatively with the MR morphology grade, which was in line with a previous study of diffusion kurtosis imaging (DKI)^30^ in SS. It also showed that parotid entropy correlated positively with the MR morphology grade. The authors speculated that as the MR morphology grade increased, heterogeneity in the microstructure and microperfusion of the parotid glands also increased^19,25–29^.

To better distinguish patients with SS from patients suspected of having SS, binary logistic regression modeling of all parameters was performed. The optimal combination of kurtosis and entropy yielded the highest AUC of 0.955. Since traditional X-ray sialography involves an invasive process with radiation, parotid MRI with an ADC texture analysis may serve as a replacement in some clinical settings.

### Limitations

There are some limitations in our study. Firstly, our sample size was small but still larger than previous studies^12,13,15^. Secondly, only X-ray sialography was used for the salivary test. Thirdly, only two *b* values were used in DWI, and the perfusion effect could not be eliminated, affecting measurement of the ADC. Fourthly, bilateral submandibular glands were not taken into consideration. Finally, the study was performed in a very homogenous setting with only one scanner in a single center. However, it is believed that these parameters could be reproduced across different scanners because the diagnostic performance of DWI did not differ significantly between 1.5-T and 3.0-T MR scanners^31,32^. Of note, according to this and previous studies, a *b* value of 1000 s/mm^2^ was recommended for DWI^12,13^.

## Conclusion

In conclusion, the present study confirmed that parotid whole-volume ADC histogram analyses, especially entropy, had great potential in diagnosing SS.

## Materials and Methods

### Participants

The ethics committee of Nanjing Drum Tower Hospital approved this study. All methods were performed in accordance with the relevant guidelines and regulations. Written informed consent was obtained from all participants. For human participants under the age of 18 years, informed consent must have been obtained from a parent and/or legal guardian. From July 2015 to December 2016, patients exhibiting xerostomia or xerophthalmia were included consecutively and prospectively based on the following criteria: (1) an initial suspected diagnosis of SS according to symptoms; (2) a willingness to undergo laboratory tests (anti-SSA and anti-SSB using enzyme-linked immunosorbent assay), ocular tests (Schirmer’s I test, positive ≤1.5 mL/15 min, and Rose Bengal staining test), labial gland biopsy of the lower lip (positive with a focus score ≥1 focus per 4 mm^2^, with one focus defined as an aggregation of ≥50 mononuclear cells), and X-ray sialography (according to Rubin and Holt scores) to establish the diagnosis; and (3) a willingness to undergo parotid MR scanning. Exclusion criteria included a medical history of radiotherapy to head and neck area, infection of hepatitis C virus (HCV), lymphoma, acquired immunodeficiency syndrome (AIDS), sarcoidosis, a history of drug use (e.g., diuretics, anticholinergic agents, and tricyclic antidepressants), and any association with other autoimmune diseases (e.g., rheumatoid arthritis or systemic lupus erythematosus). Patients were also excluded if contraindications to MR examination were present (such as cochlear or cardiac pacemaker implantation).

A total of 76 patients (67 females and 9 males; age range 17.0–74.0 years; mean age 46.4 ± 15.0 years) were enrolled. According to the AECG criteria, 52 patients (48 females and 4 males; age range 17.0–74.0 years; mean age 47.4 ± 14.8 years) were diagnosed with SS. The remaining 24 patients (22 females and 2 males; age range 17.0–68.0 years; mean 43.5 ± 15.4 years) with a suspected diagnosis of SS due to the symptoms of thirst and/or xerophthalmia did not fulfill the AECG criteria, including 8 patients with 0 positive findings, 6 patients with 1, and 10 with 2. All 76 patients had clinical symptoms of xerostomia or xerophthalmia and underwent all the listed examinations, including a serological test, Schirmer’s test, and/or the Rose Bengal test, lip biopsy, sialography, conventional imaging and DWI. Table 4 represents the clinical and laboratory information.

**Table 4:** Clinical and laboratory information of patients with Sjögren’s syndrome (SS) and patients suspected of having SS.

Tests	Patients with SS (*n* = 52)	Patients suspected of having SS (*n* = 24)
Anti-SSA and/or anti-SSB	39 (75.0)	9 (37.5)
Ocular tests	21 (40.4)	8 (33.3)
X-ray sialography	38 (73.1)	10 (41.7)
Lip biopsy	18 (34.6)	2 (8.3)

Note, The number out of bracket represents a patient number with positive findings of the test, and the number in the bracket represents the percentage.

During the same period, healthy volunteers were enrolled according to the following criteria: (1) no presentation of symptoms, signs, or history of mouth, eye, or salivary gland disease; no radiotherapy history for head or neck area, infection of HCV, AIDS, lymphoma, or sarcoidosis; and no history of drug use (e.g., diuretics, tricyclic antidepressants, and anticholinergic agents); (2) willingness to undergo serological tests (anti-SSA and anti-SSB) and Schirmer’s test; and (3) willingness to undergo parotid MR examination and a lack of contraindications. A total of 42 healthy volunteers (38 females and 4 males; age range 17.0–69.0 years; mean 45.3 ± 14.9 years) were enrolled.

### MR Examination

All participants were scanned head first on a platform of 3.0T MR scanner (Ingenia, Philips Medical Systems, Best, the Netherlands) by using a 16-channel head&neck coil in a supine position. MR scan covered the skull base to the submandibular glands, thus covering the whole volume of the bilateral parotid glands. The participants were told to refrain from swallowing during the scanning procedure.

MR sequences included axial T1-weighted imaging, T2-weighted (T2W) imaging, axial and coronal fat-suppressed T2W imaging, and DWI. The maximum gradient strength was 45 mT/s and slew rate of the MR scanner system was 200 mT/m/s. MR sequences were as follows: axial T1-weighted (T1W) turbo spin-echo sequence [parameters: repetition time (TR) = 400–675 ms; echo time (TE) = 18 ms; in-plane resolution = 0.65 × 0.75 mm^2^; field of view (FOV) = 240 × 240 mm^2^; slice thickness = 4.25 mm; intersection gap = 0.67 mm; number of signal averages (NSA) = 2; and bandwidth = 287.9 Hz/pixel]; axial T2-weighted (T2W) turbo spin-echo sequence [TR = 2500–3500 ms; TE = 90 ms; in-plane resolution = 0.45 × 0.50 mm^2^; FOV = 240 × 240 mm^2^; slice thickness = 4.25 mm; intersection gap = 0.67 mm; NSA = 2; and bandwidth = 217.6 Hz/pixel]; axial T2-weighted short tau inversion recovery (STIR) sequence [TR = 3000 ms; TE = 80 ms; in-plane resolution = 0.60 × 0.73 mm^2^; FOV = 240 × 240 mm^2^; slice thickness = 4.25 mm; intersection gap = 0.67 mm; inversion time = 200 ms; NSA = 2; and bandwidth = 228.1 Hz/pixel]; and coronal T2-weighted STIR sequence [TR = 1500–2500 ms; TE = 60 ms; acquisition matrix = 2.35 × 3.25 × 4.25; FOV = 200 × 316 mm^2^; slice thickness = 4.25 mm; intersection gap = 0.67 mm; NSA = 1; and bandwidth = 437.1 Hz/pixel]. The DWI was performed with a single-shot turbo spin-echo (TSE) sequence [*b* = 0, 1000 s/mm^2^; TR = 5711 ms; TE = 71 ms; slices = 15; slice thickness = 4 mm; slice gap = 0.93 mm; FOV = 240 × 240 mm^2^; in-plane resolution = 1.8 × 2.0 mm^2^; acquisition matrix = 132 × 120; reconstruction matrix = 256 × 256; NSA = 4; bandwidth = 819.9 Hz/pixel; flip angle (FA), 90°; shot duration = 294 ms; and acceleration factor (AF) = 2]. The diffusion time and duration of the motion probing gradient (MPG) were 39.9 and 23.1 ms, respectively, Single-shot TSE-DWI used a 180° radiofrequency refocusing pulse for each measured echo, explaining the reduction of the susceptibility artifact. The phase-encoding direction was anterior to posterior. Spectral presaturation with inversion recovery fat-suppression was used for the DWI sequence. Three motion-probing gradients along the readout, phase-encoding, and slice-selection directions were adopted. The DWI acquisition period took approximately 3 min and 48 s, and the total scan period lasted approximately 17 min and 47 s. All participants successfully underwent MRI tests without experiencing any adverse effects or discomfort.

### Image analysis

The MR images were transferred to the workstation (Extended MR WorkSpace 2.6.3.5, Philips Medical Systems, Best, the Netherlands). Two radiologists (Chen Chu, Jian He), with two and ten years of experience with head and neck radiology respectively, independently performed the measurements and interpretations independently. The radiologists were blinded to all clinical and laboratory records.

Injury degree of bilateral parotid glands was assessed separately with T1W, T2W, and T2-STIR images based on the scale developed by Makula *et al*.^11^, which reads as follows: grade 0, normal homogeneous gland parenchyma; grade 1, fine reticular or small nodular structures, nodule diameters <2 mm; grade 2, medium nodular patterns, nodule diameters between 2 and 5 mm; and grade 3, coarsely nodular, nodule diameters >5 mm. Grade 0 was treated as negative while grades 1–3 were considered as positive for diagnosing SS on MRI. A consensus was reached through discussion when divergences in the results obtained by the two radiologists occurred.

ADC maps were produced via DWI using the monoexponential model: S = S0 × exp (−*b* × ADC). DWI (*b* = 1000 s/mm^2^) presenting with the largest slice of parotid glands was adopted, and the ROI was drawn by freehand to cover a unilateral parotid gland as large as possible (area range, 176.56–738.21 mm^2^; mean, 489.08 ± 98.04 mm^2^) while maintaining a distance of 1 mm from the boundary and avoiding the retromandibular vein and external carotid artery within the gland. The ROIs were automatically copied to the ADC maps, and mean ADC value within the ROI was calculated. The mean value for bilateral parotid glands was the obtained.

A whole-volume ADC histogram analysis was performed by using an in-house software (Image Analyzer 2.0, China) as described in our previous studies^24,33^. The ROIs were drawn by freehand to cover the parotid gland as large as possible on each DWI slice (*b* = 1000 s/mm^2^). The ROIs were automatically copied to the ADC maps. After selecting all ROIs of a unilateral parotid gland (slice number: 5–10; mean: 7 ± 1), a volume of interest (VOI) was composed (volume range, 5236.76–28343.65 mm^3^; mean, 11756.34 ± 4962.54 mm^3^) to calculate the following parameters with following formulas, where *X* indicates the set of all ADC values, *N* is the number of sampled ADC pixels,$\,\bar{X}$ is the mean of *X*, and P(*i*) is the frequency of voxels with intensity *i* divided by *N*.

(i) ADC_mean_ is the mean value of all ADC values within the VOI, $\frac{1}{N}\sum _{i}^{N}X(i)$;
(ii) Skewness is histogram asymmetryaround the mean,$\,\frac{\frac{1}{N}{\sum }_{i=1}^{N}{(X(i)-\bar{X})}^{3}}{{(\sqrt{\frac{1}{N}{\sum }_{i=1}^{N}{({\rm{X}}({\rm{i}})-\bar{X})}^{2}})}^{3}}$;
(iii) Kurtosis is a measurement of histogram sharpness, $\frac{\frac{1}{N}{\sum }_{i=1}^{N}{(X(i)-\bar{X})}^{4}}{{(\frac{1}{N}{\sum }_{i=1}^{N}{(X(i)-\bar{X})}^{2})}^{2}}$;
(iv) Entropy is the distribution of gray levels over the VOI, $-\sum _{i=1}^{{N}_{l}}P(i){\mathrm{log}}_{2}P(i)$.

Measurements made by each radiologist were separately recorded for interobserver agreement analysis. The averaged value of the two measurements was calculated as the final value for each subject. One of the radiologists (Chen Chu) repeated all measurements one month later to perform intraobserver agreement analysis.

No obvious artifacts were found on DWI owing to quality control and quality assurance. Measurements were performed in all of the patients because of the lack of too much diffusion distortion. Single slices were not excluded from the whole-volume analysis on any of the occasions because of distortion artifacts.

### Statistical analysis

Kolmogorov–Smirnov test confirmed the normal distribution of quantitative data (all *P* > 0.05), which were recorded as mean ± standard deviation, while qualitative data were recorded as ratios. Continuous variables were compared using an independent two-sample *t* test, and categorical variables were compared by Fisher’s test. A ROC analysis was applied to assess the diagnostic performance of parotid ADC histogram parameters. The maximal Youden index (sensitivity + specificity − 1) was calculated to establish cutoff values. A binary logistic regression analysis based on a backward stepwise selection procedure was carried out to identify independent predictors for differentiating patients with SS from patients suspected of having SS. Goodness-of-fit Hosmer–Lemeshow test was used to assess multivariate model calibration, and graphical decile group probability was determined from a calibration plot. AUCs were compared with McNeil test. The Spearman rank correlation was performed to evaluate correlation between MR morphology grade and ADC histogram parameters. Interobserver agreement for MR morphology assessment was evaluated by calculating kappa coefficient. Intraobserver and interobserver agreements for ADC parameters measurement were assessed with ICCs (0.000–0.200, poor; 0.201–0.400, fair; 0.301–0.600, moderate; 0.601–0.800, good; 0.801–1.000, excellent). We performed all statistical analyses with SPSS (version 22.0 for Microsoft Windows x64, SPSS, IL, USA). A two-tailed *P* value < 0.05 was treated as statistically significant.

## Data Availability

The datasets generated and/or analyses during the present study are available from the corresponding author on reasonable request.

## References

[1] Vitali C (2002). Classification criteria for Sjogren’s syndrome: a revised version of the European criteria proposed by the American-European Consensus Group. Ann Rheum Dis..

[2] Vitali C (1989). Parotid sialography and lip biopsy in the evaluation of oral component in Sjogren’s syndrome. Clin Exp Rheumatol..

[3] Kalk WW (2002). Parotid sialography for diagnosing Sjogren syndrome. Oral Surg Oral Med Oral Pathol Oral Radiol Endod..

[4] Saito T (1997). Salivary gland scintigraphy with 99mTc-pertechnetate in Sjogren’s syndrome: relationship to clinicopathologic features of salivary and lacrimal glands. J Oral Pathol Med..

[5] Li M (2010). Evaluation of salivary gland scintigraphy, magnetic resonance and diffusion-weighted imaging in clinical diagnosis of Sjogren’s Syndrome. African Journal Of Microbiology Research..

[6] Niemela RK (2004). Ultrasonography of salivary glands in primary Sjogren’s syndrome. A comparison with magnetic resonance imaging and magnetic resonance sialography of parotid glands. Rheumatology..

[7] Pfeiffer K (1987). Computed tomography and diagnosis of salivary gland diseases. Radiologe..

[8] Izumi M (1996). MR imaging of the parotid gland in Sjogren’s syndrome: a proposal for new diagnostic criteria. AJR Am J Roentgenol..

[9] Niemela RK, Paakko E, Suramo I, Takalo R, Hakala M (2001). Magnetic resonance imaging and magnetic resonance sialography of parotid glands in primary Sjogren’s syndrome. Arthritis Rheum..

[10] Takashima S (1991). MR imaging of Sjogren syndrome: correlation with sialography and pathology. J Comput Assist Tomogr..

[11] Makula E (2000). The place of magnetic resonance and ultrasonographic examinations of the parotid gland in the diagnosis and follow-up of primary Sjogren’s syndrome. Rheumatology (Oxford)..

[12] Ding C (2016). Diffusion-weighted MRI findings in Sjogren’s syndrome: a preliminary study. Acta Radiol..

[13] Xu X (2017). Effects of regions of interest methods on apparent coefficient measurement of the parotid gland in early Sjogren’s syndrome at 3T MRI. Acta Radiol..

[14] Roberts C (2008). Glandular function in Sjogren syndrome: assessment with dynamic contrast-enhanced MR imaging and tracer kinetic modeling–initial experience. Radiology..

[15] Su GY (2016). Feasibility study of using intravoxel incoherent motion mri to detect parotid gland abnormalities in early-stage Sjogren syndrome patients. J Magn Reson Imaging..

[16] Bhatt Neeraj, Gupta Nishant, Soni Neetu, Hooda Kusum, Sapire Joshua M, Kumar Yogesh (2017). Role of diffusion-weighted imaging in head and neck lesions: Pictorial review. The Neuroradiology Journal.

[17] Regier M (2009). Sjogren’s syndrome of the parotid gland: value of diffusion-weighted echo-planar MRI for diagnosis at an early stage based on MR sialography grading in comparison with healthy volunteers. Rofo..

[18] Cho SH (2015). Locally advanced rectal cancer: post-chemoradiotherapy ADC histogram analysis for predicting a complete response. Acta Radiol..

[19] Makanyanga J (2017). MRI texture analysis (MRTA) of T2-weighted images in Crohn’s disease may provide information on histological and MRI disease activity in patients undergoing ileal resection. Eur Radiol..

[20] Wu Zhuo, Matsui Osamu, Kitao Azusa, Kozaka Kazuto, Koda Wataru, Kobayashi Satoshi, Ryu Yasuji, Minami Tetsuya, Sanada Junichiro, Gabata Toshifumi (2015). Hepatitis C Related Chronic Liver Cirrhosis: Feasibility of Texture Analysis of MR Images for Classification of Fibrosis Stage and Necroinflammatory Activity Grade. PLOS ONE.

[21] Fox RI (2005). Sjogren’s syndrome. Lancet..

[22] Jonsson R (2011). The complexity of Sjogren’s syndrome: novel aspects on pathogenesis. Immunol Lett..

[23] Chu C (2018). Whole-volume ADC Histogram and Texture Analyses of Parotid Glands as an Image Biomarker in Evaluating Disease Activity of Primary Sjogren’s Syndrome. Sci Rep..

[24] Zhang Y (2017). Assessment of histological differentiation in gastric cancers using whole-volume histogram analysis of apparent diffusion coefficient maps. J Magn Reson Imaging..

[25] Ganeshan B, Abaleke S, Young RC, Chatwin CR, Miles KA (2010). Texture analysis of non-small cell lung cancer on unenhanced computed tomography: initial evidence for a relationship with tumour glucose metabolism and stage. Cancer Imaging..

[26] Chen W, Giger ML, Li H, Bick U, Newstead GM (2007). Volumetric texture analysis of breast lesions on contrast-enhanced magnetic resonance images. Magn Reson Med..

[27] Ganeshan B, Skogen K, Pressney I, Coutroubis D, Miles K (2012). Tumour heterogeneity in oesophageal cancer assessed by CT texture analysis: preliminary evidence of an association with tumour metabolism, stage, and survival. Clin Radiol..

[28] Ng F, Ganeshan B, Kozarski R, Miles KA, Goh V (2013). Assessment of primary colorectal cancer heterogeneity by using whole-tumor texture analysis: contrast-enhanced CT texture as a biomarker of 5-year survival. Radiology..

[29] Sidhu HS (2017). Textural analysis of multiparametric MRI detects transition zone prostate cancer. Eur Radiol..

[30] Chu C (2017). Diffusional kurtosis imaging of parotid glands in Sjogren’s syndrome: Initial findings. J Magn Reson Imaging..

[31] Shi, R. Y., Yao, Q. Y., Wu, L. M. & Xu, J. R. Breast Lesions: Diagnosis Using Diffusion Weighted Imaging at 1.5 T and 3.0T-Systematic Review and Meta-analysis. *Clin Breast Cancer* (2017). 10.1016/j.clbc.2017.06.011 28802529

[32] Azzedine B (2015). Whole-body diffusion-weighted MRI for staging lymphoma at 3.0 T: comparative study with MR imaging at 1.5 T. Clin Imaging..

[33] Meng J (2017). Histogram analysis of apparent diffusion coefficient for monitoring early response in patients with advanced cervical cancers undergoing concurrent chemo-radiotherapy. Acta Radiologica..

